# Characterization of the First Bacterial and Thermostable GDP-Mannose 3,5-Epimerase

**DOI:** 10.3390/ijms20143530

**Published:** 2019-07-19

**Authors:** Ophelia Gevaert, Stevie Van Overtveldt, Koen Beerens, Tom Desmet

**Affiliations:** Centre for Synthetic Biology, Department of Biotechnology, Ghent University, Coupure links 653, 9000 Gent, Belgium

**Keywords:** *Methylacidiphilum fumariolicum*, biocatalysis, GDP-mannose 3,5-epimerase, l-sugars, recombinant expression

## Abstract

GDP-mannose 3,5-epimerase (GM35E) catalyzes the conversion of GDP-mannose towards GDP-l-galactose and GDP-l-gulose. Although this reaction represents one of the few enzymatic routes towards the production of l-sugars and derivatives, it has not yet been exploited for that purpose. One of the reasons is that so far only GM35Es from plants have been characterized, yielding biocatalysts that are relatively unstable and difficult to express heterologously. Through the mining of sequence databases, we succeeded in identifying a promising bacterial homologue. The gene from the thermophilic organism *Methylacidiphilum fumariolicum* was codon optimized for expression in *Escherichia coli*, resulting in the production of 40 mg/L of recombinant protein. The enzyme was found to act as a self-sufficient GM35E, performing three chemical reactions in the same active site. Furthermore, the biocatalyst was highly stable at temperatures up to 55 °C, making it well suited for the synthesis of new carbohydrate products with application in the pharma industry.

## 1. Introduction

Recently, l-sugars have come into focus as they are frequently found to be key constituents of biologically relevant molecules [1,2], such as bioactive oligosaccharides [3], antibiotics [4], and clinically useful nucleosides [5], the latter being known as building blocks for anticancer and antiviral drugs. A pioneering example thereof is 3TC, an l-nucleoside analogue which is highly effective against HIV and hepatitis B [6]. Some properties that contribute to the interest of the pharmaceutical industry in l-sugars are an advanced antiviral activity, ameliorated metabolic stability, and favorable toxicological profiles [7,8]. However, apart from some exceptions (e.g., l-arabinose, l-fucose), these valuable molecules are rare in nature and are thus rather expensive in contrast to their d-enantiomers. Despite several efforts in the last decade [7,8,9,10,11], the quest for highly efficient production processes is still ongoing in order to increase the availability of l-sugars for their exploitation on an industrial scale and exploration of additional qualities.

On that account, guanosine diphosphate (GDP)-mannose 3,5-epimerase attracts attention as the enzyme catalyzes the conversion of GDP-d-mannose (GDP-Man) to GDP-l-galactose (GDP-l-Gal) and GDP-l-gulose (GDP-l-Gul) [12]. Accordingly, this biocatalyst forms a bridge between the abundant d-sugars and their rare l-counterparts and might thus contribute to the economic production of l-sugars. Indeed, this catalytic reaction paves the way for the synthesis of both l-galactose and l-gulose, and derivatives thereof. l-Gulose is known as a component of bleomycin, a glycopeptide antibiotic with antitumor properties produced by *Streptomyces verticillus* [4,13]. Actually, the sugar moiety stimulates the uptake of this drug by cancer cells [14]. Both l-gulose and l-galactose show potential as building blocks of l-nucleoside-based antiviral and anticancer medications [5,15]. Furthermore, the latter is found as a constituent of saponins and some other biopolymers [16,17]. Due to their large structural diversity, saponins manifest various biological activities and consequently, they can be applied in the food, agronomic, cosmetic, and pharmaceutical sectors [18].

The first description of GM35E dates back to 1967, when its activity was discovered in extracts of the land snail *Helix pomatia*. The enzyme particularly attracted attention as it was the first and only known instance of a double epimerization of nucleotide sugars [19]. Since then, GM35E was linked to various functions in several eukaryotic organisms, such as agar and cell wall synthesis in algae and production of polysaccharides and glycoconjugates in plants [16,20,21,22]. Yet, its role in the l-ascorbate acid (l-AA) biosynthetic pathway in plants is studied most extensively. As l-AA affects many crucial physiological processes (e.g., stress resistance and biosynthesis of secondary metabolites) and is essential to the human diet, many efforts were made to elucidate the l-AA pathway and its regulation [22,23,24]. These investigations not only contributed to the elucidation of GM35E’s physiological function in plants, but also resulted in the determination of its crystal structure and information on its mechanism [12].

**Figure 1:**
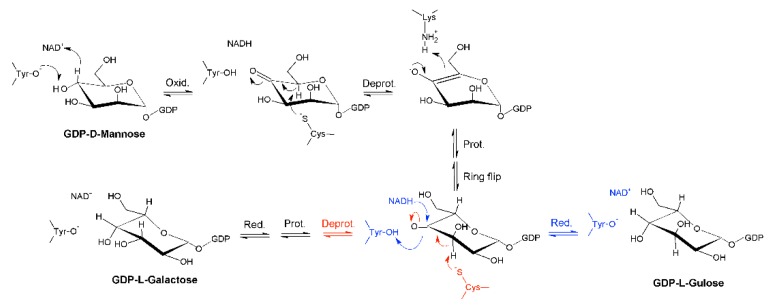
Mechanism of the GDP-mannose 3,5-epimerase. The epimerization reaction results in an equilibrium between GDP-d-mannose (GDP-Man), GDP-l-galactose (GDP-L-Gal), and GDP-l-gulose GDP-L-Gul.

Indeed, it was found that the reaction catalyzed by GM35E starts with an oxidation at C4 of GDP-mannose with the aid of an NAD-cofactor that is tightly bound to the enzyme and a conserved tyrosine residue that acts as catalytic acid to assist the deprotonation, hereby highlighting GM35E’s similarity to other enzymes in the carbohydrate epimerase family 1 (CEP1) [25], such as UDP-glucose 4-epimerases (Gal4E) [26,27]. This oxidation results in a transient keto-intermediate at C4, which merely functions to lower the p*K*_a_ of the protons on the neighboring carbons. Subsequently, a cysteine and lysine act as catalytic acid and base, respectively, and they accomplish the sequential de- and reprotonation at C5 and C3. Eventually, both the C3,5-4-keto- and C5-4-keto-epimer can be reduced back, resulting in GDP-l-galactose and GDP-l-gulose as by-product [12], as shown in Figure 1. On that account, GM35E is an exceptional epimerase as it is able to perform three distinct catalytic reactions (oxidation, epimerization, and reduction) in the same active site. This is in contrast to other nucleotide sugar pathways that require multiple enzymes for an equivalent series of reactions. Indeed, GDP-4-keto-6-deoxy-d-mannose-3,5-epimerase-4-reductase, for example, only performs epimerization and reduction. Consequently, that enzyme is dependent on a preceding oxidation by a 4,6-dehydratase [28]. Another example is the production of l-rhamnose from d-glucose, which requires consecutive oxidation at C4 and C6, epimerization at C3 and C5, and final reduction at C4. In bacteria, the latter reaction requires three separate enzymes [29]. However, it was discovered that *Arabidopsis thaliana* combines the epimerase and reductase activity within one bifunctional enzyme [30]. These findings, together with the realization that no bacterial GM35Es are characterized to date, could imply that some bacteria distribute the 3,5-epimerization of GDP-Man over several enzymes.

The present paper discusses the production and characterization of the first bacterial GM35E deriving from *Methylacidiphilum fumariolicum* strain SolV. This thermostable variant displays an optimal temperature and pH of 60 °C and 7.5 and is hypothesized to play a role in the lipopolysaccharide biosynthesis of the organism. Based on its properties, the biocatalyst is thought to be highly suitable for the industrial-scale production of l-sugars and derivatives.

## 2. Results

### 2.1. Identification of a Bacterial GM35E Homologue

To date, only GM35E enzymes from plants have been characterized; the one from *Arabidopsis thaliana* being the most extensively explored example [12,20,21,22,23]. Previous studies on GM35E all focused on the physiological role of this enzyme, principally in the l-ascorbate acid biosynthetic pathway. However, GM35E might also be considered as a significant route towards the economical and ecological production of l-sugars. Accordingly, in the interest of industrial exploitation of this C5-epimerase, the availability of bacterial homologues might be profitable. Indeed, bacterial enzymes generally display high catalytic activity and facilitate recombinant expression in prokaryotes. In addition, enzymes deriving from thermophilic bacteria are presumed to be more (thermo)stable, a property that is highly valuable as this results in a higher tolerance for the harsh process conditions practiced in industry [31]. For instance, the commonly applied high process temperatures to prevent microbial contamination, support substrate and product solubility, counteract viscosity of the reaction broth and improve the process speed [32].

The increasing amount of genomic data that has become available in the past few years allows straight-forward mining in search of bacterial homologues. UniProt contains 18 unreviewed bacterial sequences annotated as ‘GDP-mannose 3,5-epimerase’, mostly belonging to the *Proteobacteria, Actinobacteria,* and *Acidobacteria*. Of these sequences, the one from *Methylacidiphilum fumariolicum* strain SolV (*Mf*GM35E, UniProt: I0K0X9) displays the highest sequence identity (50.5%) with the GM35E gene of *Arabidopsis thaliana* (*At*GM35E, UniProt: Q93VR3). Moreover, *Methylacidiphilum fumariolicum* is a thermoacidophilic methanotroph found in volcanic environments, potentially yielding stable enzymes [33]. Consequently, *Mf*GM35E was selected for further characterization.

### 2.2. Optimization of Activity Testing

Recombinant expression of *Mf*GM35E in *Escherichia coli* was optimized and after purification, about 40 mg/L enzyme was recovered, as shown in Figure S1. Subsequently, an initial examination of the activity of *Mf*GM35E demonstrated the presence of GDP-Man together with two products of which the concentrations increased in time until an equilibrium ratio of 80:15:5 was reached. This ratio is in accordance with the conversion profile of GDP-Man towards GDP-l-Gal and GDP-l-Gul by *At*GM35E [12]. This demonstrated that the recombinant enzyme was active and was able to catalyze the same epimerization reaction as *At*GM35E.

**Figure 2:**
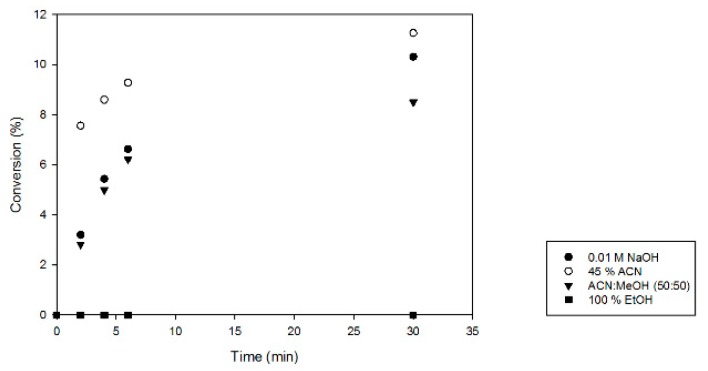
Evaluation of enzyme inactivation methods. Reaction mixtures (2 mM GDP-Man, 0.01 mg/mL MfGM35E, pH 8) were incubated at 37 °C. At defined time points, samples were 10× diluted in various inactivation solutions (0.01 M NaOH, 45% acetonitrile (ACN), acetonitrile/methanol (MeOH) (50:50), or 100% ethanol (EtOH)). The y-axis represents the conversion of GDP-Man towards GDP-L-Gal.

Next, the enzyme inactivation method for activity tests with *Mf*GM35E was optimized. As *Mf*GM35E is supposed to be a thermostable variant, only chemical methods were considered, as shown in Figure 2. Using 100% ethanol (EtOH) for inactivation resulted in degradation of the products, as was reported previously [34]. Furthermore, it became apparent that the samples inactivated in 45% acetonitrile (ACN) displayed a higher conversion towards GDP-l-Gal, suggesting that the enzyme was not inactivated immediately and thus shows some solvent resistance towards 45% ACN. Moreover, the enzyme might even be activated by the addition of solvent at this suboptimal temperature, as has previously been observed with sucrose phosphorylase [35]. The other approaches share the same efficiency. As inactivation in 0.01 M NaOH results in a high pH which might degrade NDP-sugars [36], the method of choice for full inactivation of the enzyme was acetonitrile/methanol (50:50). This optimization formed the basis for further characterization of *Mf*GM35E, revealing a specific activity of 0.47 (± 0.03) U/mg.

### 2.3. Optimal Temperature, pH, And Kinetic Properties

**Figure 3:**
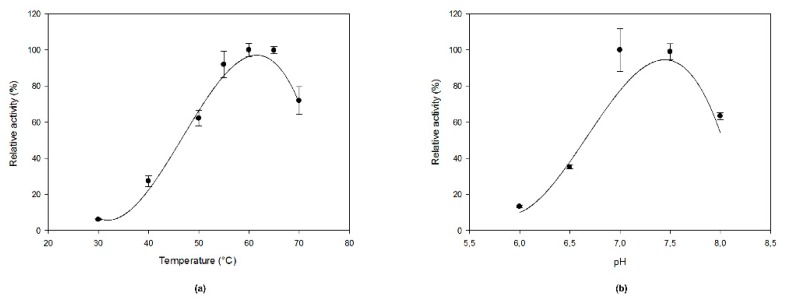
The effect of (**a**) temperature and (**b**) pH on MfGM35E activity. The pH and temperature profiles were determined in the presence of 2 mM GDP-Man at 40 °C and pH 8, respectively.

Even though *Methylacidiphilum fumariolicum* is an acidophilic organism, *Mf*GM35E showed the highest activity at pH 7.0–7.5 as it is an intracellular enzyme, as shown in Figure 3b. Its optimal temperature was 60 °C, which is at the higher end of the organism’s optimal growth temperature range of 50–60 °C, as shown in Figure 3a. The enzyme displayed at least 60% of its maximal activity between 50 and 70 °C. This is a high value in comparison to the conventional optimal temperatures of enzymes deriving from plants (e.g., around 25 °C for GM35E from *Oryza sativa* (*Os*GM35E)) [22]. Interestingly, *Mf*GM35E displayed a melting temperature (*T_m_*) of about 57 °C, whereas for *At*GM35E this was only 36 °C, as shown in Figure S2. Furthermore, the kinetic stability of *Mf*GM35E was assessed by measuring its residual activity after incubation at its optimal temperature of 60 °C and at 50 °C, an industrially relevant temperature which provides a balance between stability of both enzyme and NDP-sugars on the one hand, and the advantages of working at an elevated temperature on the other hand. Only 18% activity was retained after incubating at 60 °C for 1 h, whereas still 87% and even 63% activity was left after respectively 1 h and 4 h of incubation at 50 °C. This high kinetic stability is very promising for industrial exploitation of the bacterial variant.

Subsequently, *Mf*GM35E’s kinetic parameters for GDP-Man were determined. The enzyme exhibited Michaelis–Menten kinetics at the tested substrate concentrations, and *K_M_* and *k_cat_* values of 98 µM and 0.2 s^−1^ were deduced. Its catalytic efficiency (*k_cat_*/*K_M_*) equaled 2.04 s^−1^ mM^−1^, which is comparable to the efficiencies of *At*GM35E (9.1 s^−1^ mM^−1^) and *Os*GM35E (4.26 s^−1^ mM^−1^) [22,23]. On the one hand, the *K_M_* value is higher than the values displayed by *At*GM35E and *Os*GM35E, being 5 and 7 µM, respectively [22,23]. This might be justified by a different physiological role of GM35E in plants and bacteria. Indeed, in the latter, GM35E is hypothesized to play a role in antibiotic and lipopolysaccharide (LPS) biosynthesis (see Discussion), whereas the enzyme was detected to serve as a critical regulatory enzyme in the l-AA biosynthetic pathway in plants [37]. This potentially results in a higher affinity of plant GM35Es for GDP-Man. On the other hand, *Mf*GM35E’s *K_M_* value is in line with other related, bacterial enzymes such as GDP-mannose 4,6-dehydratase (280 µM) and GDP-l-fucose synthase (38.6 µM) [38,39].

### 2.4. Sequence and Structure Analysis of MfGM35E

**Figure 4:**
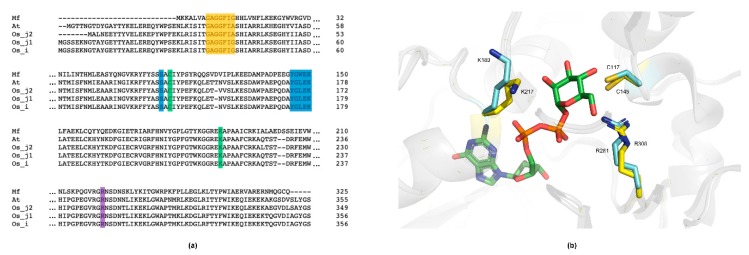
Sequence and structural analysis of MfGM35E. (**a**) Multiple sequence alignment of the GM35Es from *Methylacidiphilum fumariolicum* (Mf), *Arabidopsis thaliana* (At), *Oryza sativa* subsp. *japonica* (Os_j), and *Oryza sativa* subsp. *indica* (Os_i). Yellow, GxxGxxG motif; blue, YxxxK motif and upstream serine (S); green, catalytic cysteine (C) and lysine (K); purple, conserved arginine (R). (**b**) Representation of the active site of MfGM35E (blue) and AtGM35E (yellow). The catalytic C and K are highlighted, as well as the conserved R. GDP-mannose is colored by element (green).

**Figure 5:**
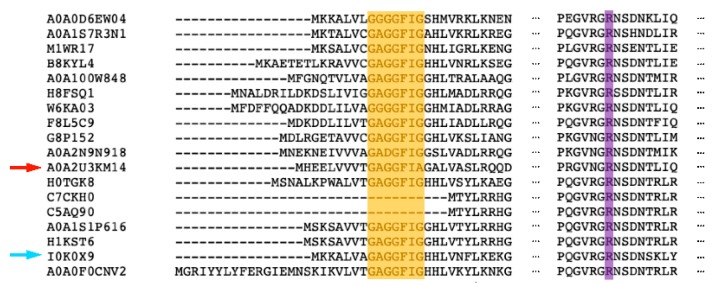
Multiple sequence alignment of the 18 putative bacterial GM35Es found in UniProt. The GxxGxxG motif is highlighted in yellow, whereas the conserved arginine (R) is colored in purple. The variants are represented by their UniProt code. The bacterial GM35E displaying the alternative GxxGxxA motif is marked by a red arrow, and *Mf*GM35E is indicated by a blue arrow.

**Figure 6:**
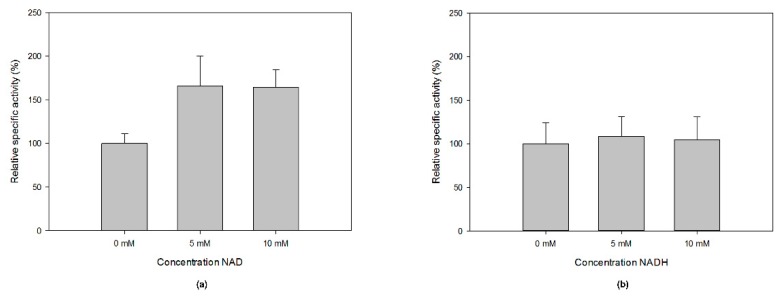
The effect of additional (**a**) NAD^+^ and (**b**) NADH on the specific activity of MfGM35E.

*Mf*GM35E is part of the short-chain dehydrogenase/reductase (SDR) superfamily, of which the members display conserved features such as a GxxGxxG motif for NAD(P)^+^ binding within an N-terminal Rossmann fold and a YxxxK catalytic motif at their C-terminus [40]. This was confirmed by a multiple sequence alignment of *Mf*GM35E and the four reviewed plant GM35Es, all sharing about 50% sequence identity with the bacterial epimerase, as shown in Figure 4a. In addition, the upstream serine as part of the catalytic triad and the catalytic cysteine and lysine, responsible for oxidation/reduction at C5 and C3 in GM35E, are highlighted. For plants, it was found that some species have an altered NAD-binding motif (GxxGxxA), which is the case for *At*GM35E for instance, as shown in Figure 4a [22,23]. Interestingly, the GM35E sequence of *Candidatus Sulfotelmatobacter kueseliae* (UniProt: A0A2U3KM14) also contains this modified GxxGxxA motif, as shown in Figure 5. The GxxGxxG motif in members of the SDR superfamily is known to tightly bind an NAD(P)^+^ molecule, obviating the need for additional NAD(P)^+^. However, it was found for *At*GM35E and *Os*GM35E that the addition of NAD^+^ resulted in an increased activity, regardless of the conservation of the GxxGxxG motif [22,23]. For *Mf*GM35E as well, a 65% increase in activity was observed, independent of the added concentration of NAD^+^, as shown in Figure 6. These findings are surprising in view of the observations with other members of the SDR superfamily and the fact that *At*GM35E’s crystal structure contains a bound NAD^+^ molecule [12]. On the contrary, supplementation with NADH had no significant impact, as shown in Figure 6.

Interestingly, during the mechanistic quest of Major et al. for the catalytic acid and base in *At*GM35E, R306 was first considered as potential acid [12]. Even though its position does not allow proton abstraction, they found that an R306A mutant displayed significantly reduced activity. Consequently, it was hypothesized that a positively charged R306 would stabilize the thiolate form of the later appointed catalytic cysteine (C145), decreasing the p*K*_a_. A multiple sequence alignment demonstrated the conservation of the arginine among both plant and bacterial GM35Es, as shown in Figure 4a and Figure 5. Moreover, also the position and orientation of the arginine (R281) with reference to the catalytic cysteine is maintained, as can be deduced from the homology model of *Mf*GM35E, as shown in Figure 4b. Consequently, these findings support a possible role of this arginine in GM35E’s reaction mechanism. In conclusion, the similarities between the active sites of bacterial and plant GM35Es suggest that enzymes deriving from both taxonomic domains employ the same reaction mechanism. Further mutational investigations might be performed to confirm this hypothesis.

## 3. Discussion

Although various applications of rare sugars are known to date, further investigation of their properties for potential use in diverse industrial sectors is still required. As a consequence of their low natural abundance, they need to be synthesized through (bio)chemical production processes in order to promote their scientific exploration and commercial exploitation. Lately, epimerases are brought into focus for the production of these valuable compounds [25]. This enzyme class is in particular promising as they can establish shortcuts in conversion processes, e.g., C5-epimerases which can form a bridge between d- and l-hexoses [41].

Accordingly, this paper presented the characterization of the first bacterial GM35E from the thermoacidophilic organism *Methylacidiphilum fumariolicum* strain SolV. This biocatalyst entails the efficient synthesis of GDP-l-Gal and GDP-l-Gul from GDP-Man, thereby combining three catalytic reactions in a single active site. This is a special feature with respect to bacteria as it was already shown that bacteria tend to split complex catalytic reactions over several enzymes whereas plant enzymes immediately combine these reactions into one enzyme [28,29]. The characterized bacterial homologue displays a very high stability and optimal temperature as compared to the GM35Es that are available today. In addition, the enzyme exhibits some solvent resistance as it was not immediately inactivated in 45% of ACN. This property was linked to thermostability already and is of interest as organic solvent systems are sometimes preferred in industry, e.g., for the glycosylation of certain pharmaceuticals [35,42]. Indeed, these systems can result in an amplified solubility of hydrophobic substrates or in the inhibition of water-dependent side reactions [43]. Moreover, the use of an organic co-solvent might alter an enzyme’s specificity or selectivity and improve its stability and activity [44,45,46]. Consequently, it might be interesting to further explore solvent engineering of *Mf*GM35E [47].

Next to the biochemical characterization of *Mf*GM35E, the occurrence of GM35E in bacteria raises the question what role the enzyme might play in these organisms, and in *Methylacidiphilum fumariolicum* in particular. Studies on the biosynthetic pathway of the anticancer compound bleomycin, which contains l-gulose as a building block, revealed the presence of blmG, a sugar epimerase which was hypothesized to catalyze the conversion of NDP-d-mannose to NDP-l-gulose [13]. A BLAST search with the respective sequence (UniProt: Q9FB21) exposed 42% sequence identity with *At*GM35E, fortifying the assumption that this concerns a GM35E. Furthermore, the activity of GM35E was recently described in the biosynthetic pathway of the nucleoside antibiotic A201A found in *Marinactinospora thermotolerans* [48]. This antibiotic contains an l-galactose moiety in its furanose configuration which is essential for the bioactivity of this drug. From these findings, it can be deduced that bacterial GM35Es feasibly play a role in antibiotic biosynthesis. On the other hand, l-sugars in general have been described as components of lipopolysaccharides (LPS), potentially influencing the organism’s virulence. This principally concerns deoxy amino l-sugars, which are attractive targets for vaccine development as these structures are specific for bacteria and do not appear in eukaryotes [49,50]. An example thereof is 2,6-dideoxy-2-acetamidino-l-galactose, a component of the O-antigen of LPS of *Pseudomonas aeruginosa* O12 and *Escherichia coli* O145 [51]. The synthesis of this compound also depends on the activity of epimerases [52]. Investigation of the genomic context of *Mf*GM35E implies involvement in LPS biosynthesis as well, as shown in Figure S3. Indeed, a gene encoding a phosphatidylglycerolphosphate synthase (UniProt: I0K0X5) was found in close proximity to the GM35E gene. Phosphatidylglycerol, an amphiphilic lipid, is known to be a basic component of bacterial membranes [53,54]. The presence of a glycosyltransferase (UniProt: I0K0Y0) next to *Mf*GM35E and a nearby methyltransferase (UniProt: I0K0Y2) supports this hypothesis [55,56].

From an application-oriented point of view, the epimerization reaction of *Mf*GM35E gives access to two expedient NDP-sugars, namely GDP-l-Gal and GDP-l-Gul. Today, an efficient production process for these activated sugars is lacking, resulting in the scarce availability and a high price (€8580/100 mg, Carbosynth 2019) of the former and the commercial absence of the latter. Recently, the production of GDP-l-galactose from l-galactose was established by Ohashi et al. in a single step reaction catalyzed by the promiscuous l-fucokinase/GDP-l-fucose pyrophosphorylase [57]. High conversion yields (92%) were obtained, however, the use of l-galactose as starting material significantly drives up production costs. The exploitation of *Mf*GM35E for production on a preparative scale would allow the synthesis of about 15 g/L GDP-l-Gal and 5 g/L GDP-l-Gul starting from 100 g/L GDP-Man. Subsequently, the recovery of the nucleotide sugars might be realized by an anion exchange chromatography protocol [58,59]. Interestingly, the availability of these NDP-sugars forms a gateway to glycorandomization, a process in which the carbohydrate moiety of antibiotics is altered in order to tailor their pharmacological properties and/or biological activity [60,61,62]. As l-galactose and l-gulose are two l-sugars that are known to be present in natural antibiotics, they form an interesting starting point for this technique [4,48]. Moreover, the l-Gul moiety in bleomycin facilitates its uptake by cancer cells, assuming that it might be interesting to link this l-sugar to other anticancer drugs as well [14]. Interestingly, most glycosyltransferases catalyze irreversible reactions, which means that coupling these enzymes with epimerases would push the epimerization reaction towards product formation, thereby increasing yields. Eventually, an integrated pathway from cheap substrate towards highly valuable compounds might be obtained as the biocatalytic production of GDP-mannose starting from mannose was recently established by various research groups [63,64].

In conclusion, this paper highlighted GM35E from *Methylacidiphilum fumariolicum* strain SolV as an industrially attractive biocatalyst with a huge potential in the production of valuable l-sugars and derivatives. Moreover, this project emphasizes the significance of epimerases for rare sugar synthesis in general.

## 4. Materials and Methods

### 4.1. Materials

All chemicals were obtained from Sigma-Aldrich (Saint Lois, MO, USA) or Carbosynth (Compton, UK) and were of the highest purity, unless stated otherwise.

### 4.2. Gene Cloning And Transformation

The codon optimized GM35E genes from *Methylacidiphilum fumariolicum* strain SolV (UniProt: I0K0X9) and *Arabidopsis thaliana* (UniProt: Q93VR3) were synthesized and subcloned into the pET21 vector at *Nde*I and *Xho*I restriction sites, providing a C-terminal His_6_-tag, by GeneArt Gene Synthesis (Thermo Fisher Scientific, DWaltham, MA, USA). The constructs were transformed in *E. coli* BL21(DE3) electrocompetent cells for protein expression.

### 4.3. Enzyme Production And Purification

In this study, 250 mL lysogeny broth (LB) medium (10 g/L trypton, 5 g/L yeast extract, and 5 g/L NaCl) supplemented with 100 µg/mL ampicillin was inoculated with 2% (*v*/*v*) stationary culture and incubated at 37 °C and 200 rpm till the OD_600_ equaled 0.6. Subsequently, cultures were cooled down to 20 °C and enzyme production was induced by the addition of isopropyl β-d-thiogalactopyranoside (IPTG) to a final concentration of 1 mM (overnight incubation at 20 °C and 200 rpm). Cells were harvested by centrifuging for 30 min at 9000 rpm and 4 °C. The obtained pellets were frozen and stored at −20 °C for at least 1 h. For enzyme extraction and purification, each pellet of a 250 mL culture was resuspended in 8 mL of lysis buffer (500 mM NaCl, 10 mM imidazole, 100 µM phenylmethane sulfonyl fluoride (PMSF) and 1 mg/mL lysozyme in 50 mM sodium phosphate buffer pH 7.4) and cooled on ice for 30 min. Next, the cells were subjected to 3 times 2.5 min of sonication (Branson sonifier 250, level 3, 50% duty cycle). Cell debris was collected by 15 min of centrifugation at 12,000 rpm and 4 °C. The supernatant, containing the soluble protein fraction, was subsequently filtered through a 0.2 µm polyethersulfone membrane filter (VWR, Leuven, Belgium). Next, the His_6_-tagged proteins were purified to apparent homogeneity by Ni-NTA chromatography, with small variations to the supplier’s description (Thermo Fisher Scientific, Waltham, MA, USA). The resin was washed twice with 8 mL wash buffer (500 mM NaCl, 20 mM imidazole in 50 mM sodium phosphate buffer pH 7.4). Protein was eluted with 3 times 4 mL elution buffer (500 mM NaCl, 250 mM imidazole in 50 mM sodium phosphate buffer pH 7.4). As a final step, buffer was exchanged to 50 mM HEPES pH 8 by using Amicon Ultra-15 centrifugal filter units with 30 kDa cut-off (Merck Millipore Darmstadt, Germany). About 40 mg/L of enzyme was recovered.

The protein content was determined by measuring the absorbance at 280 nm with the NanoDrop2000 Spectrophotometer (Thermo Fisher Scientific, Waltham, MA, USA). The extinction coefficient and molecular weight of His_6_-tagged GM35Es were calculated using the ProtParam tool on the ExPASy server (https://web.expasy.org/protparam/). Molecular weight and purity of the protein were verified by sodium dodecyl sulfate polyacrylamide gel electrophoresis (SDS-PAGE; 12% gel). The enzyme’s electrophoretic behavior corresponded well with its predicted molecular mass of about 37 kDa.

### 4.4. Optimization of Product Analysis and Enzyme Inactivation

First, the separation of NDP-sugars using high performance anion exchange chromatography and pulsed amperometric detection (HPAEC-PAD; Dionex ICS-3000 system, Thermo Fisher Scientific, Waltham, MA, USA) was addressed. Based on product formation by *At*GM35E (2 mM GDP-Man and 1 mg/mL purified enzyme in a total of 200 µL 50 mM HEPES pH 8.0 at 21 °C), it was found that GDP-Man, GDP-l-Gal, and GDP-l-Gul could be separated by an isocratic method using a mixture of 850 mM NaOAc and 100 mM NaOH for 20 min.

Secondly, the enzyme inactivation method was evaluated. Reaction mixtures contained 2 mM GDP-Man and 0.1 mg/mL *Mf*GM35E in a total volume of 500 µL 50 mM HEPES pH 8.0. Reactions were incubated at 30 °C and 1000 rpm. Then, 30 µL of the reaction mixture was inactivated in 270 µL inactivation solution (0.01 M NaOH, 50% acetonitrile, acetonitrile/methanol (50:50), or 100% ethanol).

### 4.5. Activity Testing and Enzyme Characterization

The enzymatic activity was assayed by the converting activity towards GDP-l-Gal. For all activity tests, samples were taken at defined time points and inactivated in a mixture of acetonitrile and methanol (50:50) unless stated otherwise. Afterwards, samples were analyzed on the Dionex ICS-3000 system as described in Section 4.4.

For the initial examination of *Mf*GM35E’s activity, 2 mM GDP-Man, 1 mM NAD, and 1 mg/mL purified enzyme were incubated in 50 mM HEPES pH 8.0 at 21 °C (200 µL total reaction volume). Samples were inactivated in 0.01 M NaOH. The represented specific activity was determined with 2 mM GDP-Man and 0.1 mg/mL purified enzyme in a total reaction volume of 500 µL 50 mM HEPES pH 8.0 at 50 °C. The influence of pH on enzyme activity was evaluated in the presence of 2 mM GDP-Man, McIlvain buffer pH 6.0–8.0 at 40 °C, and the optimal temperature was determined with 2 mM GDP-Man in 50 mM HEPES pH 8.0 (500 µL total reaction volume). The enzyme’s kinetic stability was examined by incubating purified protein (0.04 mg/mL) at 50 and 60 °C in 100 mM MOPS pH 7.5. Then, the residual activity was measured (2 mM GDP-Man, 100 mM MOPS pH 7.5, 55 °C) and compared to the activity of untreated enzyme. For the kinetic parameters, the enzyme’s activity incubated with various substrate concentrations (20–2000 µM) was evaluated in 100 mM MOPS pH 7 at 60 °C. For each condition, appropriate enzyme concentrations (0.04–1 mg/mL) were employed. The effect of NAD(H) was checked with 2 mM GDP-Man, 0.05 mg/mL purified enzyme in a total volume of 500 µL 100 mM MOPS pH 7.5 at 50 °C. The kinetic parameters, including the Michaelis–Menten constant (*K_M_*) and turnover number (*k_cat_*), were determined using a Michaelis–Menten plot created in SigmaPlot (Systat Software, San Jose, CA, USA).

### 4.6. Thermal Shift Assay

The melting temperature of the proteins was measured using differential scanning fluorimetry (DSF) in a CFX Connect 96 machine (Bio-Rad, Hercules, CA, USA). Briefly, 5 µg of purified enzyme was mixed with 10 µL 1/400 SYPRO Orange in a total volume of 25 µL 50 mM HEPES pH 8.0. All samples were loaded in triplicate in a 96-well plate and sealed afterwards. The fluorescence was measured while increasing the temperature 1 °C/min from 20 °C till 95 °C. The fluorescence resonance energy transfer (FRET) option was chosen to allow excitation at 450 to 490 nm and emission at 560 to 580 nm.

### 4.7. Sequence Analysis

Bacterial homologues of GM35E from *Arabidopsis thaliana* were found through a basic local alignment search tool analysis at the UniProt server using default parameters (https://www.uniprot.org/blast/). All necessary sequences were retrieved from the UniProt database (https://www.uniprot.org). Multiple sequence alignments were performed with Clustal Omega using default parameters (https://www.ebi.ac.uk/Tools/msa/clustalo/) [65]. The Ensembl Bacteria web interface (https://bacteria.ensembl.org/index.html) was used to browse the genomic region surrounding MfGM35E.

### 4.8. Homology Modelling

YASARA was used to generate a homology model of *Mf*GM35E using default parameters. The crystal structure of the *Arabidopsis thaliana* GM35E served as template (Protein Data Bank ID: 2C5A, 2C54, 2C5E, and 2C59). Figures were made with PyMOL v2.0.

## Supplementary Materials

The following are available online at https://www.mdpi.com/1422-0067/20/14/3530/s1, Figure S1: SDS-PAGE of *Mf*GM35E purification; Figure S2: Melting curves obtained through DSF; Figure S3: Genomic context of GM35E in *Methylacidiphilum fumariolicum* strain SolV.

## Abbreviations

GM35E	GDP-mannose 3,5-epimerase
Gal4E	UDP-glucose 4-epimerase
CEP	carbohydrate epimerase
GDP-Man	GDP-d-mannose
GDP-l-Gal	GDP-l-galactose
GDP-l-Gul	GDP-l-gulose
l-AA	l-ascorbic acid
LPS	lipopolysaccharide
SDR	short-chain dehydrogenase/reductase
ACN	acetonitrile
EtOH	ethanol
MeOH	methanol
LB	lysogeny broth
IPTG	isopropyl β-d-thiogalactopyranoside
PMSF	phenylmethane sulfonyl fluoride
DSF	differential scanning fluorimetry
FRET	fluorescence resonance energy transfer
