# Supplementary material for: Characterization of the First Bacterial and Thermostable GDP-Mannose 3,5-Epimerase
Source: Int J Mol Sci. 2019 Jul 19;20(14):3530. doi: 10.3390/ijms20143530 (PMC6678494; doi:10.3390/ijms20143530)
Supplement: Supplementary file 1 [file ijms-20-03530-s001.zip › Figure S1.pdf]

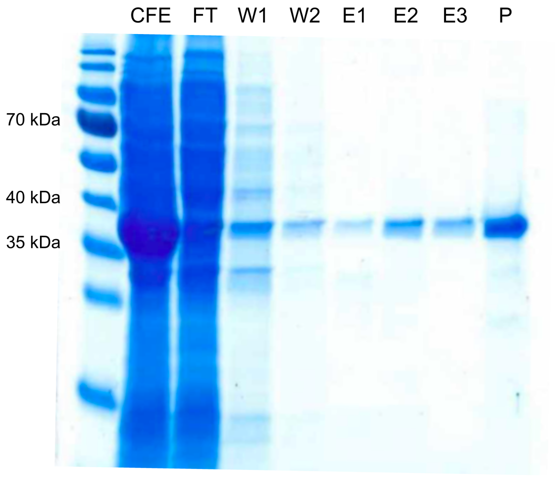

Figure S1. SDS-PAGE of *Mf*GM35E purification. CFE: cell free extract; FT: flow through; W: wash fraction; E: eluted fraction; P: pure enzyme (after buffer exchange).
