# Supplementary material for: Characterization of the First Bacterial and Thermostable GDP-Mannose 3,5-Epimerase
Source: Int J Mol Sci. 2019 Jul 19;20(14):3530. doi: 10.3390/ijms20143530 (PMC6678494; doi:10.3390/ijms20143530)
Supplement: Supplementary file 1 [file ijms-20-03530-s001.zip › Figure S3.pdf]

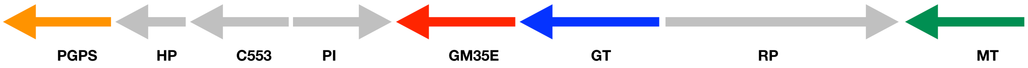

Figure S3. Genomic context of GM35E in *Methylacidiphilum fumariolicum* strain SolV. The surrounding genes are a phosphatidylglycerophosphate synthase (PGPS), conserved exported hypothetical protein (HP), cytochrome c553 (C553), intracellular proteinase inhibitor (PI), glycosyltransferase (GT), outer membrane receptor protein (RP) and Rsm22 family methyltransferase (MT).
