# Supplementary figures and images for: Characterization of the First Bacterial and Thermostable GDP-Mannose 3,5-Epimerase
Source: Int J Mol Sci. 2019 Jul 19;20(14):3530. doi: 10.3390/ijms20143530 (PMC6678494; doi:10.3390/ijms20143530)

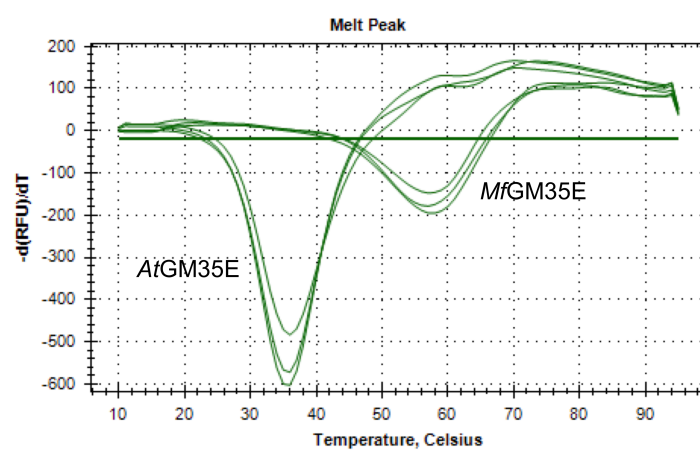

**Figure S2.** Melting curves obtained through DSC. The analysis was performed in triplicate.

Supplement: Supplementary file 1 [file ijms-20-03530-s001.zip › Figure S2.pdf]
